# Case report: Recurrent cervical spinal stenosis masquerading as myalgic encephalomyelitis/chronic fatigue syndrome with orthostatic intolerance

**DOI:** 10.3389/fneur.2023.1284062

**Published:** 2023-11-30

**Authors:** Charles C. Edwards, Charles C. Edwards, Scott Heinlein, Peter C. Rowe

**Affiliations:** ^1^Department of Pediatrics, Division of Adolescent and Young Adult Medicine, Johns Hopkins University School of Medicine, Baltimore, MD, United States; ^2^Maryland Spine Center, Mercy Medical Center, Baltimore, MD, United States; ^3^Lifestrength Physical Therapy, Inc., Towson, MD, United States

**Keywords:** cervical stenosis, myalgic encephalomyelitis/chronic fatigue syndrome, postural tachycardia syndrome, orthostatic intolerance, case report

## Abstract

**Introduction:**

Myalgic encephalomyelitis/chronic fatigue syndrome (ME/CFS) is a complex, chronic, multi-system disorder that is characterized by a substantial impairment in the activities that were well tolerated before the illness. In an earlier report, we had described three adult women who met criteria for ME/CFS and orthostatic intolerance, and had congenital or acquired cervical spinal stenosis. All three experienced substantial global improvements in their ME/CFS and orthostatic intolerance symptoms after recognition and surgical treatment of the cervical stenosis. After a several year period of improvement, one of the individuals in that series experienced a return of ME/CFS and orthostatic intolerance symptoms.

**Main symptoms and clinical findings:**

Radiologic investigation confirmed a recurrence of the ventral compression of the spinal cord due to a shift of the disc replacement implant at the involved cervical spinal level.

**Therapeutic intervention:**

Decompression of the spinal cord with removal of the implant and fusion at the original C5-C6 level was once again followed by a similar degree of improvement in function as had been observed after the first operation.

**Conclusion:**

This recapitulation of the outcomes after surgical management of cervical stenosis provides further evidence in support of the hypothesis that cervical spinal stenosis can exacerbate pre-existing or cause new orthostatic intolerance and ME/CFS. Especially for those with refractory symptoms and neurological signs, surgical interventions may offer relief for selected patients with this complex condition.

## Background

Myalgic encephalomyelitis/chronic fatigue syndrome (ME/CFS) is a complex, chronic, multi-system disorder that is characterized by a substantial impairment in the activities that were well tolerated before the illness, leading to a reduction in health-related quality of life (1–7). Other prominent symptoms include profound fatigue, unrefreshing sleep, post-exertional worsening of symptoms (known as post-exertional malaise, PEM), orthostatic intolerance, and cognitive dysfunction. Orthostatic intolerance is identified in up to 96% of adolescents and up to 90% of adults with ME/CFS (1, 5, 8–10). Post exertional malaise--which can be provoked by increased physical or cognitive activity, by orthostatic stress, and by neural strain--can persist for hours, days, or weeks (1, 11–13). Cognitive problems in ME/CFS include difficulty with attention, short-term memory, and processing, and often are worsened during periods of increased exhaustion (1, 14). The mechanism of symptom generation for ME/CFS as a whole is unknown, and there is no uniformly effective pharmacologic therapy. The COVID-19 pandemic has led to an increased prevalence of individuals meeting criteria for ME/CFS as part of their post-acute COVID syndrome (also known as “long COVID”) (15, 16).

Current treatment of ME/CFS is symptomatic and supportive. Several groups have emphasized the association between anatomic abnormalities of the cervical spine or the skull base and ME/CFS or fibromyalgia, a condition characterized by chronic widespread pain and fatigue (17–21). We previously reported a case series of three adult women who had congenital or acquired cervical spinal stenosis (22). All met criteria for ME/CFS. After one-or two-level cervical decompression and disc replacement, all three patients experienced substantial global improvements in their ME/CFS and orthostatic intolerance. We now report that one of the individuals in that series experienced a several year improvement before a return of ME/CFS symptoms. Radiologic investigation confirmed a shift of the disc replacement implant at the involved cervical spinal level, leading to a recurrence of the ventral compression of the spinal cord. Subsequent decompression of the spinal cord was once again followed by a similar degree of improvement in function as had been observed after the first operation. This recapitulation of the outcomes after surgical management of cervical stenosis provides further evidence in support of the hypothesis that cervical spinal stenosis can exacerbate pre-existing or cause new onset ME/CFS and orthostatic intolerance.

## Methods

The clinical history and examination methods for this individual were described in detail in the original case series [she was patient 3 in the 2018 publication (22)]. Improvements in health-related quality of life before and after cervical disc replacement surgery were assessed using (1) a clinician-assigned Karnofsky Performance score (possible range 0–100) (23) and (2) the self-reported Medical Outcomes Study 36-item Short Form Health Survey (SF-36) physical function (PF) subscale score (possible range 10–30) (24), a Wellness score (ranging from 0 to 100, with 0 meaning dying and 100 meaning the best one could imagine feeling) (25), and the Wood Mental Fatigue Inventory (ranging from 0 to 36) (26). Higher scores indicate better function on the Karnofsky, SF-36, and Wellness score, while higher scores on the Wood indicate worse cognitive function. The Institutional Review Board of the Johns Hopkins Medical Institutes allows for reporting of clinic data without informed consent if those data were collected as part of routine care.

## Case report

### Symptom onset

*Patient 3* from the 2018 study was healthy and active, working full time until fatigue and lightheadedness developed abruptly at age 31 following international travel. There had been no associated head or neck trauma. She reported episodic nausea, lightheadedness, slurred speech, and blurred vision. A lumbar puncture was normal. Head-up tilt testing to investigate the orthostatic symptoms showed a baseline supine blood pressure of 128/64 with a heart rate of 64 beats per minute (bpm). After being positioned upright to 70 degrees, her heart rate increased to 76 bpm at 10 min. After 25 min upright, she developed syncope and was unresponsive, with a junctional bradycardia (heart rate 45 bpm) and an undetectable blood pressure, consistent with neurally mediated (or reflex) syncope.

She stopped working after 4 months of symptoms, mainly due to persistent fatigue despite normal amounts of sleep. Other symptoms included PEM, right shoulder and neck pain, difficulty with concentration, headaches, nausea, a decrease in appetite, anxiety, and lightheadedness. Lightheadedness was present any time she attempted to rise from a seated position. She had enough cognitive dysfunction and intermittent somnolence that she felt uncomfortable driving. Reading was impaired, and she could not complete crossword puzzles as before. She had impaired name and word recall, and confusion when completing small tasks such as pouring laundry detergent in the appropriate place. She developed PEM after just one brief errand in a day, and required a wheelchair for longer trips out of the house. She reported an overall Wellness score of 35/100. She was minimally responsive to medical, psychiatric, and physical therapy management over a period of 7 years.

### Recognition and surgical treatment of cervical spinal stenosis

**Figure 1:**
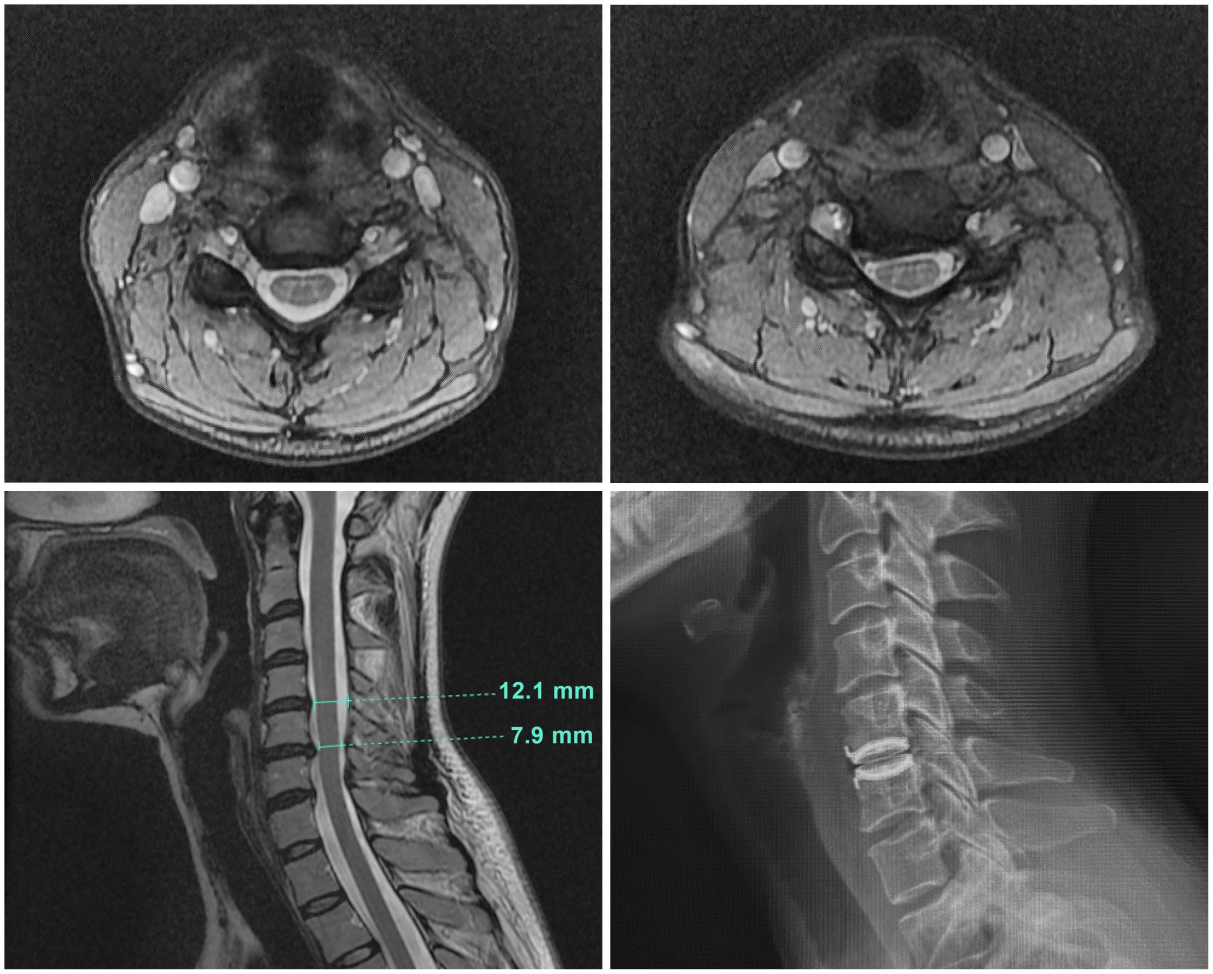
Cervical spine MRI images. Top left: A transverse image at C4-5 showing normal spinal canal diameter. Top right: A transverse image at C5–6 illustrating the site of cord compression with asymmetrical indentation on the right side of the cord. Bottom left: The sagittal cervical cord MRI image showed a broad right paracentral disc bulge at C5-6 causing mild cord compression and canal stenosis, with an antero-posterior (AP) cervical canal diameter of 7.9 mm at that level. The cervical canal diameter at the radiographically normal level of C4-5 was 12.1 mm. Bottom right: The plain radiograph of the sagittal cervical spine illustrating the original decompression at C5-6 and disc replacement with a mechanical artificial disc implant.

Abnormal physical examination findings evident early in the course of the illness included 2-3+ deep tendon reflexes, a fine resting tremor, and a positive Hoffman sign bilaterally. At the 7-year point, further investigation of the Hoffman sign with a cervical spine MRI (Figure 1) showed a bulging disc at C5-6 that caused asymmetric compression of the cervical spinal cord, with a spinal cord antero-posterior diameter variously measured as between 7.9–8.5 mm, consistent with cervical canal stenosis. On examination by a spine specialist (CCE II), she had right wrist weakness, and some loss of sensation in the median nerve distribution on that side.

### Surgical technique for operation 1

The bulging C5-6 disc was the principal source of spinal cord compression and thus removal of the offending bulging disc was required. Reconstructive options were disc replacement versus fusion. The surgeon’s recommendation and the patient’s preference was for disc replacement. The perceived benefits of disc replacement over fusion were: motion preservation, easier recovery, lower risk of adjacent disc degeneration over time, and the avoidance of fusion-related complications such as nonunion.

Decompression at C5-6 and disc replacement proceeded in the standard fashion as described previously (22). Briefly, a one-inch transverse incision was made in a skin crease to the left of midline on the anterior neck. Using standard techniques under microscopic visualization, the C 5–6 disc annulus, nucleus pulposus, endplate cartilage and posterior longitudinal ligament were removed with a combination of manual curettes and high-speed burr. Posterior projecting endplate osteophytes were thinned and removed. All soft-tissues (disc herniation and fibrocartilage) projecting dorsal to the posterior vertebral endplate were removed so as to thoroughly expand the spinal canal and decompress the spinal cord. The posterior portions of uncovertebral joints were thinned and removed to provide generous space for the exiting C6 nerve roots.

Reconstruction was achieved with placement of a mechanical artificial disc implant (Bryan – Medtronic) under fluoroscopic guidance. Postoperative x-rays demonstrated the implant to be well positioned within the C5-6 intervertebral space (Figure 1).

### Operation 1 recovery

She reported resolution of tremor, headache, and right shoulder and neck discomfort in the first post-operative week. Over the next 2 months, she tolerated gradual increases in exercise. Symptom-free standing tolerance improved to 45 min. At 3 months, she was able to participate in intermediate downhill skiing. At 7 months of recovery, she was able to engage in vigorous exercise without developing PEM. Two years after surgery, she sustained a tear in the anterior cruciate ligament (ACL) during a fall while skiing, but recovered from surgery without difficulty. Her cognitive improvements allowed her to resume reading complex texts; word-finding problems were no longer present. She was able to resume driving, cooking, shopping errands, and other normal daily tasks.

### Recrudescence of ME/CFS symptoms

Two years after the ACL repair, and 4 years after the original cervical spine procedure, she experienced a gradual re-emergence of fatigue, which limited her participation in physical activities. Subsequent yoga was limited by the inability to maintain arm-overhead poses. Driving required a lowered hand position holding the steering wheel and the patient reported sensations of discomfort with upper limb extension. She noted more fatigue and nausea when physically active, such as with skiing, and she had more falls on ski slopes. Recovery periods following activities increased in duration as fatigue worsened. She had a return of cognitive dysfunction, and myalgias in the limbs, the upper back, and the lower back, along with difficulty with lateral neck rotation, numbness in the upper limbs, and decreased sensitivity to temperature of the fingertips on her left side. Despite adequate amounts of sleep (up to 12 h nightly), she awakened unrefreshed. Lightheadedness and blurred vision became more frequent.

To address symptoms and exam findings that were consistent with neurogenic thoracic outlet syndrome (TOS), she returned to physical therapy, which transiently helped the upper limb symptoms, but fatigue and PEM persisted. TOS symptoms resurfaced over the next year, and could be aggravated by simply wearing a backpack. She experienced periodic numbness in her right hand as well as right shoulder pain. Her energy levels diminished further, and she reported increased lightheadedness and heart rate when squatting and standing. She required a motorized wheelchair to travel longer distances. Hand grip weakness increased, with notable impairment on the right side. Sensory testing suggested facial hypoesthesia in lower two thirds of her face, and hyperesthesia to pinprick in the right arm and leg.

### Recurrent cervical stenosis and its treatment

**Figure 2:**
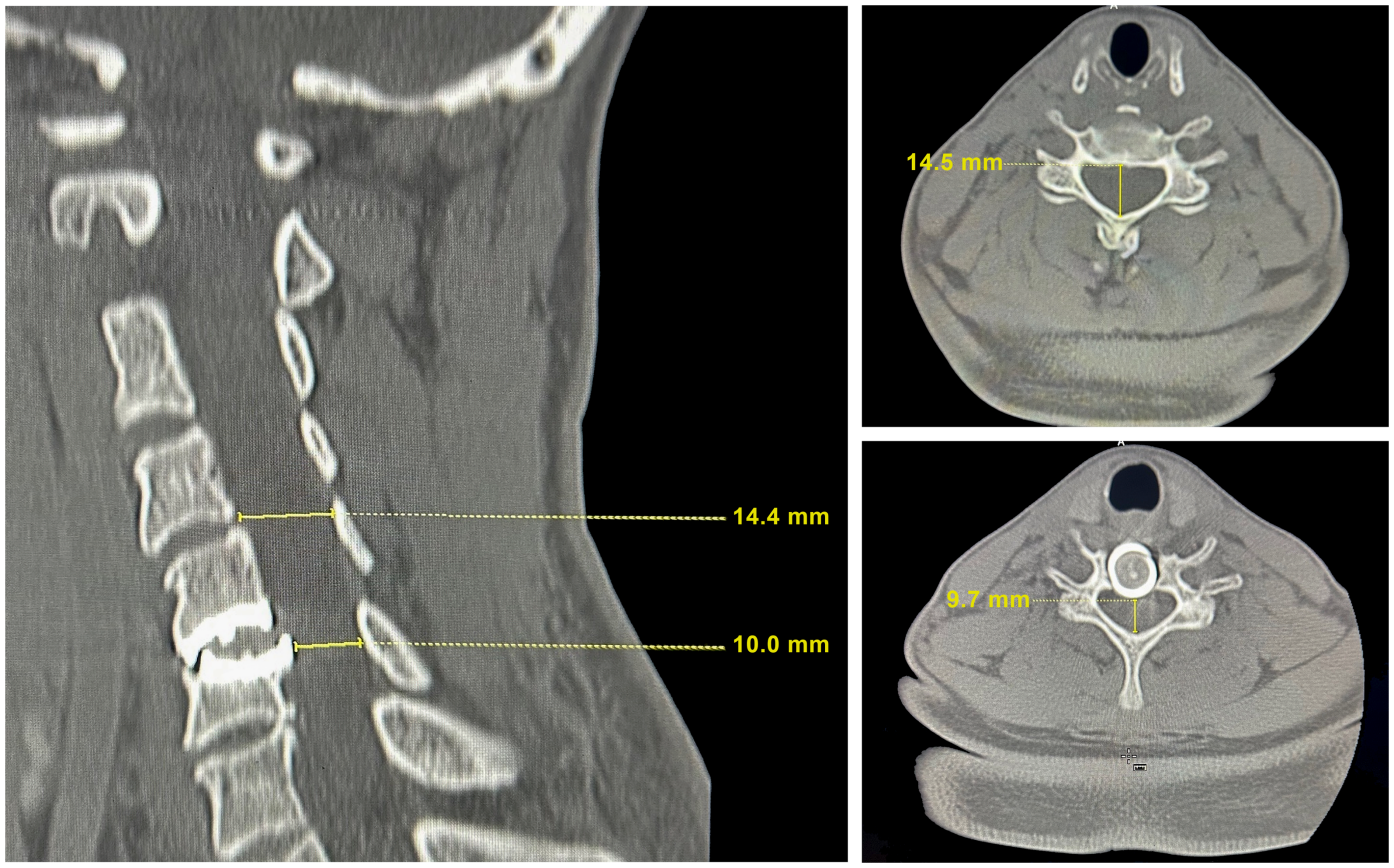
CT scan images at the time of recrudescence of symptoms. Left: The sagittal cervical cord image showing degradation of the mechanical disc with protrusion into the spinal canal. The mechanical disc indents the C6 vertebrae resulting in a reduction of the vertebral height compared to adjacent vertebrae and to the height in Figure 1. Top right: A transverse image at C4-5 with a cervical canal diameter for radiographically imaged hard tissue measured at 14.4–14.5 mm, which is within the range of normal. Bottom right: A transverse image at C5-6 illustrating the site of cord compression resulting from the mechanical disc shift into the spinal canal.

A repeat CT scan of the cervical spine, however, showed a several millimeter shift of the C6 implant plate dorsally compared to the radiographs taken after the first operation, causing recurrent spinal cord compression (Figure 2). Compared to the radiographically normal level of C4-5, the C6 implant shift caused a 33.1% reduction in the cervical canal diameter. There was diminished height of the C6 vertebral body. Bone density was normal. We therefore speculated that the posterior shift of the disc implant had occurred as a consequence of trauma from falls during skiing, and that recurrent cervical spinal stenosis was contributing to her worsening symptoms. She underwent the removal of the original disc replacement implant at C5-6, revisional decompression at C5-6, and placement of an interbody arthrodesis at that level.

### Surgical technique for operation 2

**Figure 3:**
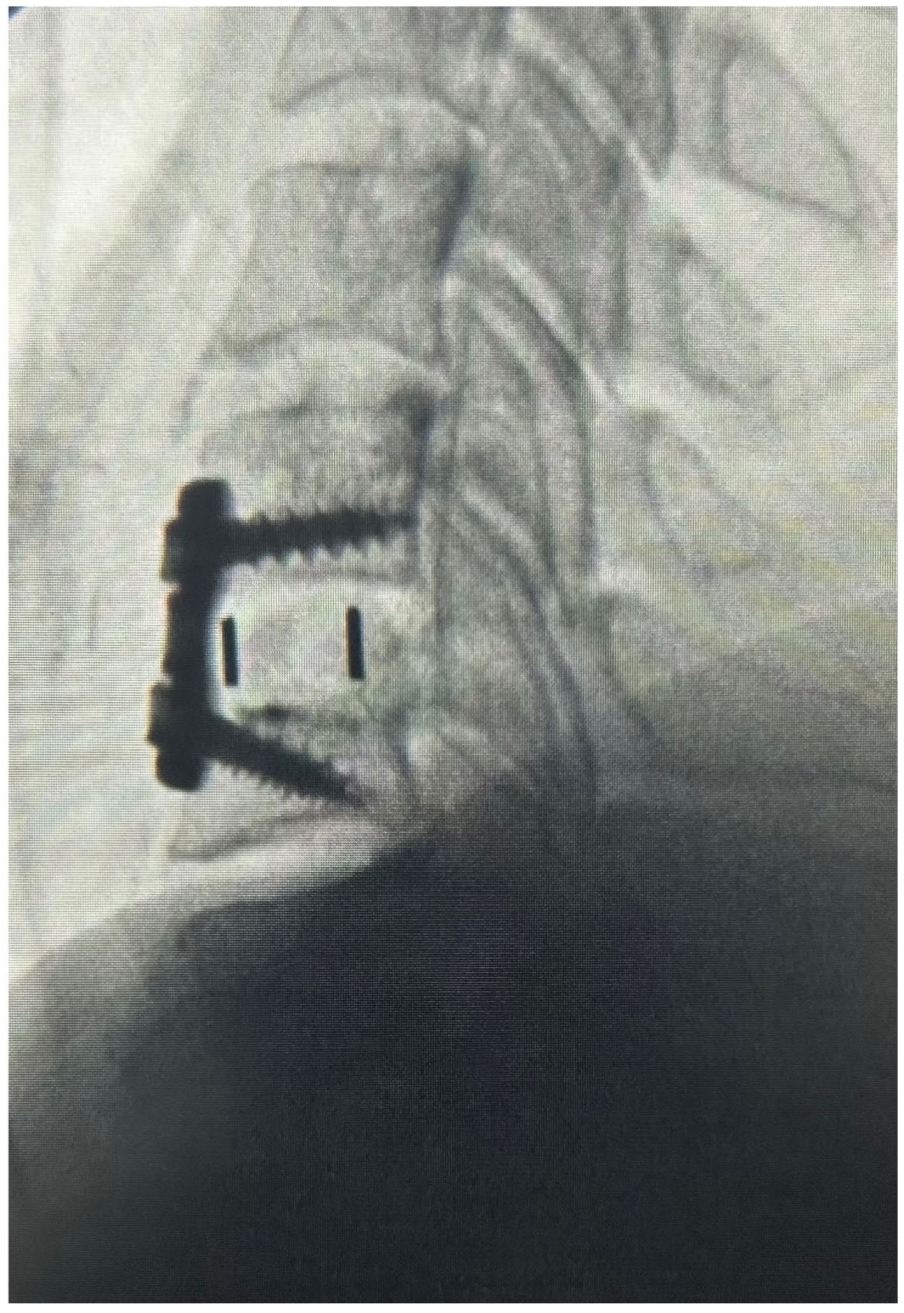
The x-ray image of the sagittal cervical spine after operation #2 illustrating the decompression at C5-6, disc replacement with a PEEK strut, and fusion.

Under general anesthesia, the previous incision was opened and the anterior aspect of the spine exposed. The interfaces between the vertebral endplates and the implant were developed using a microcurrette and an osteotome. The implant was removed. Fibrocartilage that had formed lateral to the implant was scrapped away with microcurrettes so as to restore the space within the spinal canal and foramen for the spinal cord and C6 nerve roots, respectively. With the revision decompression complete, a PEEK strut (Calvary Spine) of the appropriate dimension was selected to restore the intervertebral height. The central portion of the strut was filled with a combination of Infuse bone morphogenic protein (Medtronic) and Grafton demineralized bone matrix (Medtronic) to promote fusion. The strut was placed into the intervertebral space and the segment was stabilized with an Atlantis plate and screws (Medtronic) (Figure 3).

### Operation 2 recovery

**Figure 4:**
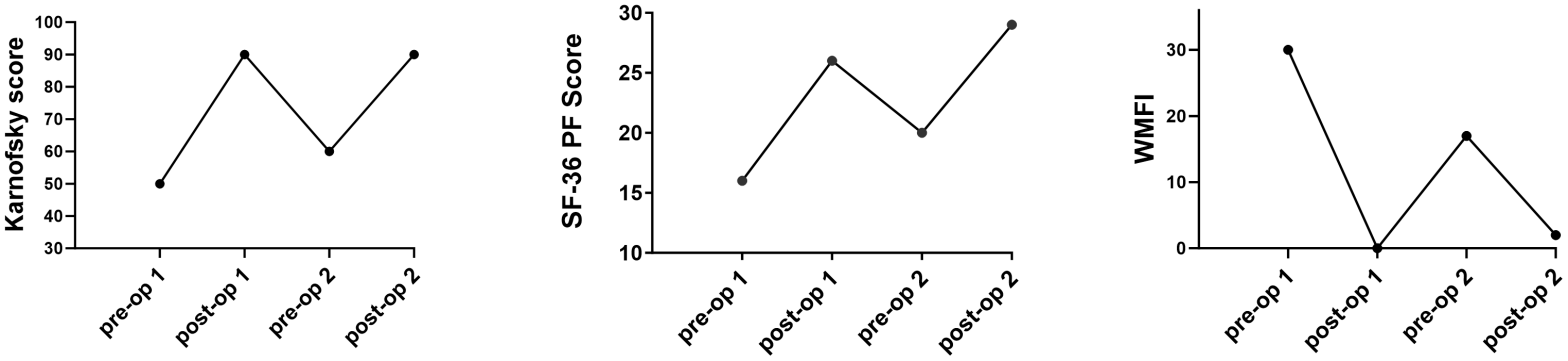
Pre- and post-operative scores on the Karnofsky, SF-36 Physical Function subscale, and the Wood Mental Fatigue Inventory.

At the 2 months post-operative visit, she reported increased energy levels and was fully functional with activities of daily living, without radicular symptoms. By 6 months after surgery, she reported complete resolution of neck and arm pain, as well as improvement in lightheadedness and in her other ME/CFS symptoms. She tolerated daily one-mile walks, prepared three meals a day, and was re-engaged in creative mental activities. Physical examination documented normal upper limb muscle strength, as well as normal symmetric sensation to light touch. Figure 4 illustrates the changes in the quality of life questionnaire scores before and after each surgical procedure, extending to the 9-month follow-up point after the second surgery.

## Discussion

This case report describes a woman with a seven-year history of disabling ME/CFS symptoms who was eventually discovered to have single-level cervical spinal stenosis. After recognition and surgical management of cervical spinal stenosis, she experienced a near complete improvement in function, along with resolution of her orthostatic tachycardia. Post-operatively, she was able to engage in vigorous physical activities. Following several years of improvement, however, she noted the insidious return of her main ME/CFS symptoms. Conservative management again was ineffective. Ultimately, imaging identified a shift in the C5-6 disc implant, which was causing recurrent contact with the ventral cervical spinal cord, and was associated with concordant findings of cervical myelopathy.

Removal of the shifted disc replacement, and revision of the C5-6 decompression, this time with fusion, was again followed by a prompt improvement in overall activity and a return to more normal function. She experienced a significant improvement in myelopathic symptoms, lightheadedness, post-exertional malaise, and anxiety levels. As seen in Figure 4, there was a striking improvement in the SF-36 PF, Karnofsky Performance, and Wood Mental Fatigue Inventory scores. These outcomes add to the growing body of evidence that compression of the cervical spinal cord can provoke symptoms of orthostatic intolerance and ME/CFS, either through direct injury to the ventral cervical spinal cord, or through interruption of neural pathways.

Our findings emphasize the importance of careful neurologic examination of patients with ME/CFS. Heffez and colleagues reported a high prevalence of neurologic exam abnormalities and findings consistent with cervical myelopathy or Chiari malformation among referred patients with fibromyalgia (18). Among those who underwent surgical treatment of Chiari malformation or cervical spinal stenosis, there was a significant improvement in symptoms at the 1 year follow-up point, but no notable improvement in those in the non-surgical comparison group (18).

The cervical spinal stenosis observed in our patient is analogous to spinal cord compression occurring in individuals with joint hypermobility and atlanto-axial instability (AAI). Henderson and colleagues observed that excessive rotational movement at C1-2 could be associated with symptoms of headaches, neck pain, hand numbness, lower extremity numbness, arm weakness, syncope, and pre-syncope (21). These symptoms improved significantly upon alignment, fusion, and stabilization of C1-2 (21).

Several groups have hypothesized that neuro-inflammation involving microglial activation is important in the pathogenesis of ME/CFS. Microglial activation can be provoked by acute trauma or collective stressors shifting microglial cells to a pro-inflammatory phenotype, increasing cytokines, neurotoxic factors, and chemokines (27–30). With peripheral nerve injury or trauma to the spinal cord, microglial inflammation can persist or worsen, producing CNS dysfunction (27–29). If the PNS and CNS are already sensitized by microglial inflammation in ME/CFS, the addition of cervical spinal cord compression observed in this series would likely result in heightened symptoms and sensation compared to non-ME/CFS individuals (29).

We caution that surgical treatment is not indicated for all individuals with ME/CFS or with bulging discs. Careful selection of patients for surgery is needed. Disc degeneration is a part of the normal ageing process (31). Bulging discs can be asymptomatic. Boden and colleagues report that 19 percent of asymptomatic individuals demonstrate spinal abnormalities using magnetic resonance imaging (32). Furthermore, asymptomatic spinal abnormalities are observed more in study participants age 40 and greater than in individuals younger than age 40 (28% vs. 14%) (32). An investigation into the prevalence of spinal cord compression studied 1,211 adults aged 20–79 years, none of whom had a history of brain or spinal surgery, comorbid neurological diseases, severe neck pain, or symptoms related to sensory or motor disorders. Among this group, 87.6% had at least 1 mm of cervical disc protrusion posteriorly, with 5.3% having spinal canal compression (33). This study reported that a significant degree of spinal cord compression could be tolerated in otherwise healthy individuals without any symptoms, again emphasizing the necessity to weigh overall impairment, neurologic exam findings, and radiographic results.

This study calls for greater consideration of ME/CFS when evaluating the need for surgical management. There must be radiographic findings, concordant neurological findings, and failure of conservative management in order to justify surgery. However, the increased sensitivity of neurons suggest that physicians ought to carefully examine radiographic images with the understanding and expectation that mild degrees of compression may be significant for individuals with ME/CFS. Henderson and colleagues have advised that neuroanatomic abnormalities should be considered when orthostatic intolerance symptoms are produced by neck flexion, extension, or lateral rotation (21). Furthermore, we recommend collecting dynamic radiographic images in cervical flexion, extension, and lateral rotation for patients with ME/CFS to best observe the degree of compression and movements that increase symptom onset.

As is the case for otherwise healthy individuals, those with a pre-existing diagnosis of ME/CFS or orthostatic intolerance can develop cervical stenosis over time. Without imaging from before the onset of ME/CFS symptoms, we cannot be certain whether the cervical disc degeneration in our patient was present from the outset, causing her entire clinical syndrome, or whether she had true ME/CFS and then the cord compression developed later, complicating and interfering with spontaneous improvements in the illness. The fact that our patient experienced moderate to severe impairment with relatively mild compression of the cervical spinal cord favors the latter explanation, while the robustness and completeness of the improvement would be more consistent with the former. In any event, pathological deep tendon reflexes and the onset of symptoms in association with mild spinal stenosis both call for further investigation into hypersensitivity of the spinal cord of ME/CFS patients with modest cervical myelopathy.

## Conclusion

The surgical treatment of ME/CFS symptoms due to mild cervical spinal stenosis and effacement can result in a significant improvement in a wide array of symptoms. A detailed neurological examination should be incorporated into the evaluation of every patient with ME/CFS. In light of the increased incidence of ME/CFS after COVID-19, patients with abnormal neurological examinations or refractory symptoms warrant consideration of cervical spine abnormalities which might otherwise be asymptomatic in healthy individuals. Further research is needed to explore the underlying mechanisms linking cervical spinal stenosis and ME/CFS symptoms, as well as to investigate the effectiveness of surgical intervention in larger cohorts. Nonetheless, these findings highlight the importance of considering cervical spinal stenosis as a potential cause of ME/CFS symptoms and support the concept that surgical interventions may offer relief for selected patients with this complex condition. Due to prevalent ligament laxity in ME/CFS, radiographic images documenting the spinal cord’s condition along its movements play a crucial role in proper discovery in the mechanism of symptoms.

## Data availability statement

The original contributions presented in the study are included in the article/supplementary material, further inquiries can be directed to the corresponding author/s.

## Ethics statement

The studies involving humans were approved by the Johns Hopkins Medicine Institutional Review Board. The studies were conducted in accordance with the local legislation and institutional requirements. Written informed consent for participation was not required from the participants or the participants' legal guardians/next of kin in accordance with the national legislation and institutional requirements. Written informed consent was obtained from the individual(s) for the publication of any potentially identifiable images or data included in this article.

## Author contributions

CE III: Writing – original draft, Writing – review & editing. CE II: Conceptualization, Supervision, Writing – review & editing. SH: Conceptualization, Supervision, Writing – review & editing. PR: Conceptualization, Supervision, Writing – review & editing.
